# ABC‐type diffuse large B‐cell lymphoma presenting as rotator cuff tendinopathy: A diagnostic dilemma and review of the literature

**DOI:** 10.1002/ccr3.2630

**Published:** 2020-01-20

**Authors:** Bryce David Beutler, Rohee Krishan, Pawel Parafianowicz, Mark B. Ulanja, Christie Elliott, Joel France, Raheel Islam, Nageshwara Gullapalli

**Affiliations:** ^1^ Department of Internal Medicine Reno School of Medicine University of Nevada Reno NV USA; ^2^ Department of Pathology and Laboratory Medicine Reno School of Medicine University of Nevada Reno NV USA

**Keywords:** cancer, diffuse large B‐cell lymphoma, lymphoma, rotator cuff

## Abstract

Diffuse large B‐cell lymphoma often presents with extranodal manifestations involving the musculoskeletal system. Shoulder pain is particularly worrisome for malignancy. Individuals presenting with refractory upper extremity complaints should undergo a prompt and thorough evaluation for cancer, as a delay in diagnosis can result in an unfavorable outcome.

## Graphical abstract

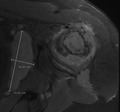

## 1 INTRODUCTION

Diffuse large B‐cell lymphoma (DLBCL) is the most common type of non‐Hodgkin lymphoma (NHL) among adults, accounting for approximately 25% of all NHLs in the United States and affecting over 18 000 individuals each year.^1^ It typically presents with prominent lymphadenopathy and/or systemic “B” symptoms—fevers, night sweats, and weight loss. However, extranodal involvement is not uncommon; it occurs in up to one‐third of patients and may involve one or multiple organ systems.^2^ A definitive diagnosis is established based on excisional tissue biopsy.

We describe a man who developed DLBCL with an atypical presentation involving the shoulder and testicles. In addition, we review the characteristics of other patients with DLBCL mimicking shoulder pathologies and discuss the differential diagnosis and treatment options for this common hematologic malignancy.

## 2 CASE REPORT

A healthy 51‐year‐old man presented to the emergency department with progressively worsening left shoulder pain of four‐month duration that had been acutely exacerbated three days earlier by a ground‐level fall. The patient reported that he first noticed a dull ache when playing golf. Radiographs obtained in the emergency department showed moderate degenerative changes but no acute abnormalities. A diagnosis of rotator cuff tendinitis was established. The patient was subsequently provided with an arm sling and discharged for outpatient follow‐up.

The patient was evaluated in clinic approximately three weeks after his initial presentation in the emergency department. He reported minimal pain relief despite daily use of the arm sling and oral naproxen sodium. Physical examination revealed significant tenderness to palpation over the left glenohumeral joint. Range of motion of the left shoulder was restricted with abduction and extension. The Hawkins‐Kennedy test and external rotation lag sign were positive. A presumptive diagnosis of rotator cuff tendinopathy vs tear was established. The patient was referred for magnetic resonance imaging (MRI) and outpatient physical therapy. However, a trial of physical therapy was required for health insurance authorization of imaging; the first session was scheduled for one week after the clinic visit.

The patient began physical therapy as planned. However, three days after his first session, he presented to the emergency department complaining of testicular swelling. An ultrasound was performed and revealed hydrocele. The patient was diagnosed with epididymitis and prescribed oral ciprofloxacin. The swelling failed to resolve after a two‐week course of antibiotics. He was subsequently evaluated by urology, who recommended continuing ciprofloxacin for eight weeks. Symptoms had partially resolved following two months of treatment.

**Figure 1:**
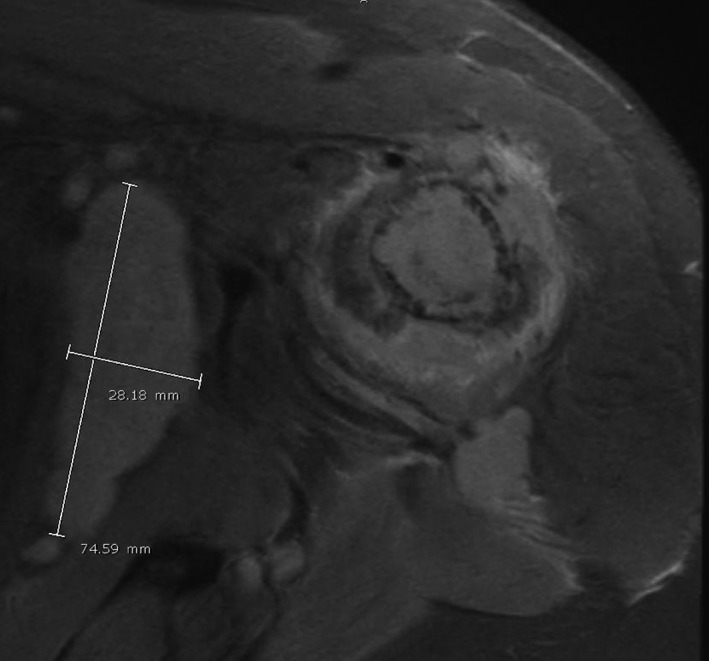
MRI of the left shoulder (coronal view) shows a large tumor replacing the marrow of the proximal humerus with an aggressive‐appearing periosteal reaction and cortical destruction. There is also a nodal mass in the left axilla anterior to the subscapularis muscle measuring approximately 6.4 × 7.5 × 2.8 cm

**Figure 2:**
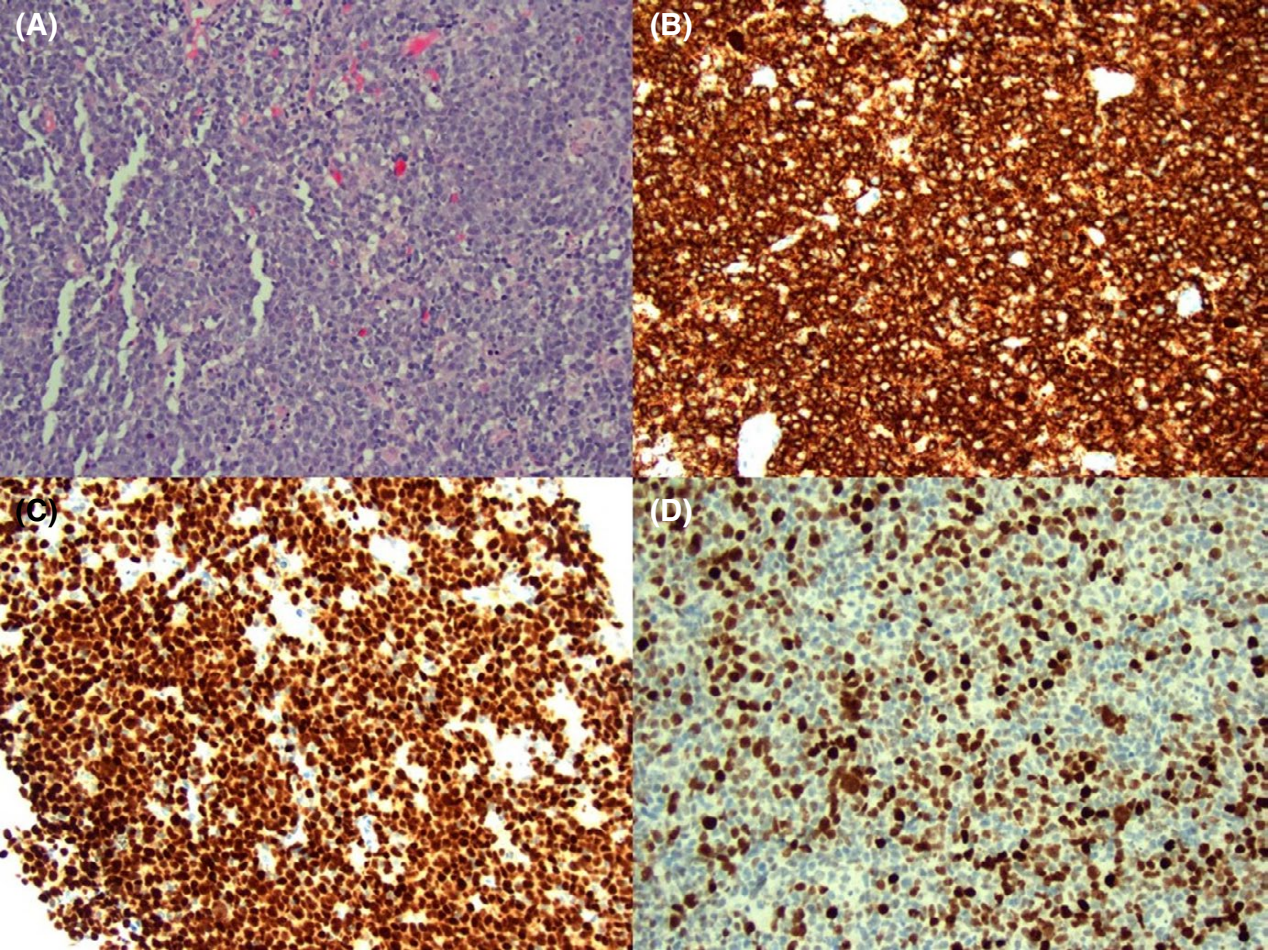
A left axillary lymph node biopsy shows a B‐cell lymphoma with high‐grade features [hematoxylin and eosin, x20] (A). Subsequent immunohistochemical analysis demonstrated diffuse positivity of CD20 (B), PAX5 (C), and Ki‐67; focal positivity of Bcl‐6 (D); and negative CD10, CD21, CD30, and Bcl‐1 markers

The left shoulder pain failed to improve after three months and twelve physical therapy sessions. An MRI without contrast was ultimately obtained and revealed severe tendinopathy of the supraspinatus tendon as well as a large tumor replacing the marrow of the proximal humerus (Figure 1). Extensive adenopathy was also noted. Four days later, the patient was seen by the hematology/oncology team; providers suspected osteosarcoma or chondrosarcoma and ordered an urgent lymph node biopsy. Computed tomography (CT)‐guided biopsy of a left axillary lymph node showed a B‐cell lymphoma with high‐grade features. The immunophenotype supported an activated B‐cell (ABC, nongerminal center) subtype. Immunohistochemical analysis demonstrated diffuse positivity of CD20, PAX5, and Ki‐67; focal positivity of Bcl‐6; and negative CD10, CD30, and Bcl‐1 markers (Figure 2). The CD21 immunohistochemical stain highlighted dendritic cell meshworks associated with follicular structures. cMYC stained less than 40% of cells, and therefore, the lymphoma was not a double expresser. The fluorescence in situ hybridization (FISH) panel was negative for a double/triple‐hit lymphoma. A definitive diagnosis of ABC‐type DLBCL was therefore established.

**Figure 3:**
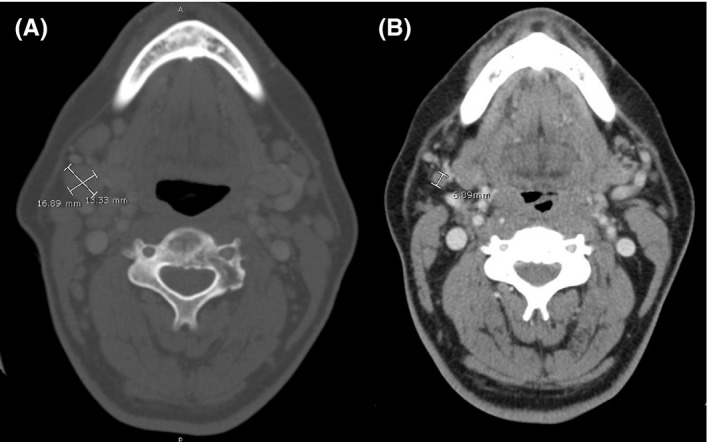
CT scan of the neck shows an enlarged right submandibular lymph node before (A) and after (B) R‐CHOP. The lymph node is significantly smaller in size after ten weeks (three cycles) of chemotherapy

**Figure 4:**
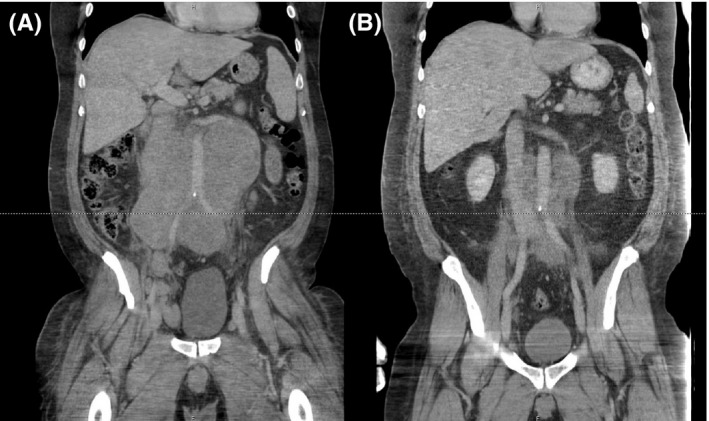
CT scans of the neck, chest, abdomen, and pelvis demonstrated marked retroperitoneal lymphadenopathy (A); this presented as testicular swelling. There is significant improvement after ten weeks (three cycles) of R‐CHOP chemotherapy (B)

CT scans of the neck, chest, abdomen, and pelvis were subsequently acquired. Notable findings included splenomegaly, an enlarged right submandibular lymph node (Figure 3A), a lytic lesion in the C3 vertebral body, and left hydronephrosis secondary to distal urethral obstruction by retroperitoneal lymphadenopathy (Figure 4A). MRI of the brain was normal, and cerebrospinal fluid analysis showed no malignant cells. Repeat scrotal ultrasound showed moderate right testicular hydrocele as well as left varicocele. Correlation of the CT and ultrasound findings indicated that the scrotal swelling represented secondary varicocele due to the retroperitoneal lymphadenopathy; therefore, testicular radiation or surgery was not pursued. The patient was started on rituximab (375 mg/m^2^), cyclophosphamide (750 mg/m^2^), doxorubicin (50 mg/m^2^), vincristine (1.4 mg/m^2^), and prednisone (R‐CHOP) every three weeks. There were no risk factors for central nervous system disease, as testicular involvement was excluded, and thus, central nervous system‐directed prophylaxis was not administered.

**Figure 5:**
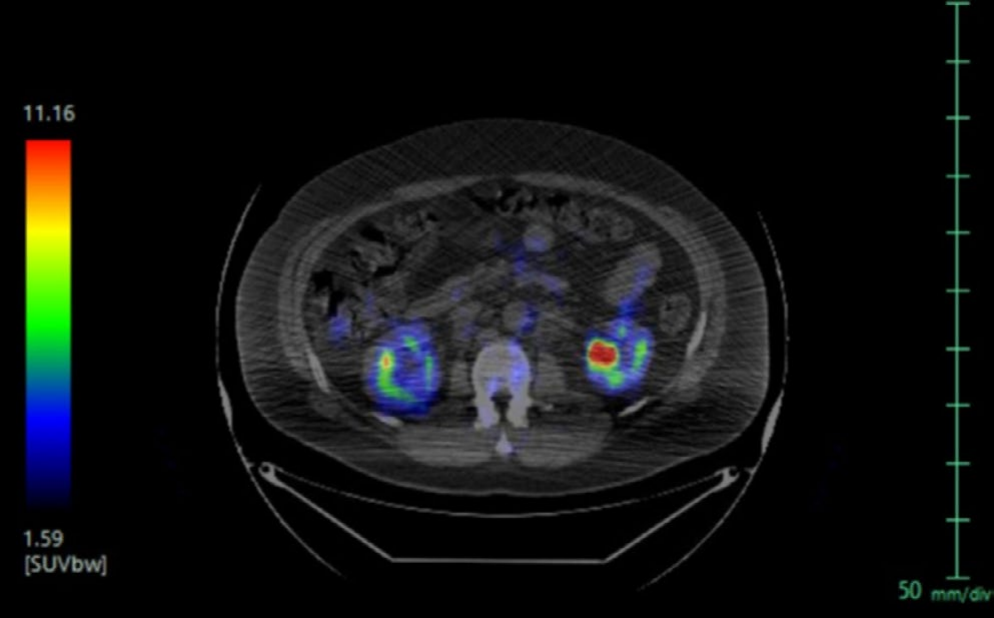
CT‐PET scan of the skull base to mid‐thigh obtained after the sixth and final cycle of R‐CHOP showed improved retroperitoneal adenopathy. The lymph nodes were FDG avid, suggestive of residual disease

The patient tolerated the therapy well. After the first cycle of R‐CHOP, the patient reported that his shoulder pain was modestly improved and that his scrotal swelling had resolved. Repeat CT scans were obtained after ten weeks and three cycles of R‐CHOP. Lymphadenopathy was markedly improved in the submandibular (Figure 3B) and retroperitoneal region (Figure 4B). The patient continued the R‐CHOP regimen for another ten weeks, completing a total of six cycles. A positron emission tomography‐computed tomography (PET‐CT) scan of the skull base to mid‐thigh was obtained after the last cycle of R‐CHOP and revealed fluorodeoxyglucose (FDG) activity of the retroperitoneal lymph nodes and left humeral lesion; imaging was otherwise normal (Figure 5). Notably, the retroperitoneal lymph nodes were decreased in size as compared to the previous CT scan. The patient subsequently began radiation therapy to the left shoulder at a dose rate of 250 centigrays (cGy) with a final external beam dose of 3750 cGy. At his most recent follow‐up, the patient had returned to work and complained only of mild, intermittent left shoulder pain.

## 3 DISCUSSION

Diffuse large B‐cell lymphoma is one of the most common hematologic malignancies in the United States, with an annual incidence of approximately 5.6 cases per 100 000 individuals. Historically, the prognosis of DLBCL was relatively poor; prior to the year 2000, the five‐year survival rate ranged from 40% to 50%. However, the approval of rituximab—a core component of the modern DLBCL treatment regimen—by the Food and Drug Administration (FDA) in 1997 has resulted in significantly improved survival. Indeed, five‐year survival increased to 60% in 2002 and has continued to climb steadily over the past two decades.^3^

DLBCL most commonly presents in middle age, with a median age of diagnosis of 66 years.^3^ The hallmark clinical feature is marked lymphadenopathy. Extranodal manifestations, however, are not uncommon and often involve the central nervous system and/or gastrointestinal tract.^4^ Systemic “B” symptoms may also occur. Less common findings include generalized fatigue, ascites, and pulmonary effusions.

Musculoskeletal complaints represent a rare but important presentation of an underlying malignancy. Interestingly, there is a particularly strong correlation between shoulder pain and cancer. A recent study by Pedersen et al demonstrated that a new diagnosis of cancer is established in approximately 1 out of every 142 patients presenting with an initial complaint of adhesive capsulitis (“frozen shoulder”).^5^ These data suggest that the suspicion for malignancy should be heightened in individuals presenting with shoulder pain and stiffness, particularly in cases in which conservative treatment fails to improve symptoms. Laboratory studies and imaging should therefore be considered in individuals with refractory shoulder dysfunction.

Malignant lymphoma of bone accounts for 7% of all bone malignancies and <5% of extranodal lymphomas.^6^ Neoplasms can often be detected on plain film radiography. The radiographic appearance can be normal, sclerotic, lytic, or mixed sclerotic‐lytic. In some individuals, DLBCL appears as an extra‐osseous soft tissue mass and cortical breach with consequent pathologic fracture.^7^ A periosteal reaction may be present and can be identified by a hallmark lamellated “onion skin” appearance; this feature portends a poor prognosis.^8^ Diffuse sclerosis of a vertebral body, also known as “ivory vertebra,” is a radiologic sign associated with osseous lymphoma. However, ivory vertebra is also associated with osteosarcoma, osteoblastic metastasis, and Paget disease.^9^

There are no truly distinctive or pathognomonic signs to reliably differentiate osseous lymphoma from primary bone tumors based on plain film radiography alone, and thus, other imaging modalities are often required to establish a diagnosis. MRI is particularly useful, as it has a high sensitivity for marrow replacement. On T1‐ and T2‐weighted sequences, DLBCL demonstrates hypo‐ and hyperintensity, respectively. However, if there is fibrosis within the tumor, T2‐weighted images may appear hypointense. Contrast‐enhanced CT (CECT) scan can also show marrow infiltration, but offers superior visualization of cortical and trabecular destruction. CECT be used for studying marrow disease and for planning a biopsy when MRI is contraindicated.^10^

A definitive diagnosis of DLBCL is established via biopsy. Histologically, DLBCL has a diffuse growth pattern with large cells that are usually five times the size of normal lymphocytes. These cells may resemble immunoblasts—characterized by amphophilic cytoplasm with one eccentric nucleus—or centroblasts with pale or basophilic cytoplasm; chromatin margination that makes the chromatin appear vesicular; and two to three nucleoli near the membrane.^11^

The classification of DLBCL is currently evolving due to increasing throughput capacity to analyze genetic material. At present, DLBCL is divided into two broad categories: double/triple‐hit lymphomas (DHLs) with specific cytogenetic rearrangements and nondouble/triple‐hit lymphomas. Nondouble/triple‐hit lymphomas are further subtyped based on the cell of origin into germinal center B‐cell‐like (GCB) and activated B‐cell‐like (ABC) tumors. The specific subtype has important clinical implications, as individuals with GCB tumors typically exhibit superior survival.^12^ The subtype is most accurately established based on an analysis of expression of fifteen genes. However, this technology is not yet widely available, and thus, immunochemical (IHC) analysis can be used to approximate the cell of origin into GCB and non‐GCB tumors.^13^ The current classification schema may not reflect the degree of genetic diversity DLBCL. Indeed, it is conceivable that new molecular targets for drug development will be identified through further genetic analyses.^14^

Our patient presented with shoulder pain that was initially diagnosed as a rotator cuff injury; DLBCL was not identified until several months after his symptoms began. Indeed, although this presentation is rare, other authors have described similar cases. In 2018, Liu et al described a 64‐year‐old woman who presented with six months of worsening right shoulder pain and limited range of motion.^15^ Plain radiographs disclosed only mild osteoarthritis. However, two months later, the patient continued to complain of progressively worsening pain. A complete physical examination was performed revealed a shoulder mass measuring 15 × 15 cm. CT and MRI of the shoulder showed expansive bone destruction extended within the cortical bone of the upper humerus and swelling of the surrounding soft tissue. Bone scintigraphy was also preformed and demonstrated signs of a malignant tumor. A definitive diagnosis of DLBCL was established with a biopsy.

A similar case was reported several years earlier by Caporale et al^16^ Authors described an 80‐year‐old man who presented with a six‐month history of continuous severe right shoulder pain and limited range of motion. Plain film radiographs showed only glenohumeral arthritis. An MRI was subsequently obtained and revealed an in‐growing inhomogeneous lesion in the anteromedial aspect of the right humeral head. Biopsy with immunohistochemical staining established a diagnosis of DLBCL.

The individuals described in the above‐referenced cases share several similarities with our patient: the clinical presentation of shoulder pain and limited range of motion; normal findings on plain film radiography; and the absence of constitutional symptoms, such as night sweats, fevers, and weight loss. All three patients were treated with R‐CHOP, which is the current standard of therapy. However, the patient described by Caporale also had surgical excision of the tumor and a reverse shoulder prosthesis due to the size of the mass. In contrast, the patient described by Liu et al exhibited a complete response to chemotherapy alone; a two‐year follow‐up showed increased range of motion, decreased pain, and no evidence of recurrent or metastatic disease.

Several other investigators have also described DLBCL presenting as shoulder pain. Characteristics of these patients are summarized in Table 1.^15^, ^16^, ^17^, ^18^, ^19^, ^20^, ^21^, ^22^, ^23^ Patients ranged in age from 30 to 80 years and most commonly presented with shoulder pain, stiffness, or both. There was a significant male gender predilection. No difference in the frequency of left vs right shoulder involvement was observed.

**Table 1:** Patients with lymphoma presenting as shoulder pain.^15^, ^16^, ^17^, ^18^, ^19^, ^20^, ^21^, ^22^, ^23^

Year	Sex	Age	Side	Presentation	Ref
2018	F	64 y	R	Right shoulder pain and limited range of motion	15
2016	M	72 y	L	Three days of neck pain radiating to the shoulder and upper back that occurred during heavy lifting	17
2016	M	50 y	L	Swelling, erythema, and recurrent of the shoulder that developed at the site of a rotator cuff repair surgery that had been performed several years earlier	18
2013	M	80 y	R	Continuous severe shoulder pain for 6 mo	16
2013	M	31 y	R	Pain and swelling of the shoulder for 6 mo	19
2011	F	53 y	L	Progressive, painful disability of the left arm	20
2003	F	70 y	L	Shoulder and arm pain of five‐month duration that was initially diagnosed and managed as bursitis	21
2002	M	75 y	L	Dull shoulder pain and limited range of motion of one‐month duration	22
1999	M	30 y	R	Fever and pain in the shoulder and inguinocrural area	23

Abbreviations: F, female; L, left; M, male; R, right; Ref, reference; y, years of age.

The considerable genetic diversity of DLBCL can be attributed to the cells of origin: Antigen‐exposed B cells found within germinal centers. These cells are exposed to an environment that promotes rapid proliferation, cytidine deamination (for receptor editing), and somatic hypermutation.^14^ The prominent molecular disturbance in the ABC subtype of DLBCL is hyperactivation of NF‐kB; this is due to a mutation of the gene *TNFAIP3*, which encodes a negative regulator of NF‐kB. Indeed, 30% of individuals who develop the ABC subtype are found to have two mutated copies of this gene. The ABC subtype of DLBCL can also be distinguished from the GCB subtype based on the *BCL‐6* gene. *BCL‐6* is required for plasma cell differentiation, and translocations in this gene are twice as common in the ABC as compared to GCB subtype.^24^

The mainstay of treatment for DLBCL for the past twenty years has been R‐CHOP, which represents the first‐line therapy per the National Comprehensive Cancer Network (NCCN) guidelines. However, the medical community is currently in the midst of a paradigm shift in the management of this common hematologic malignancy, as recently identified cytogenetic markers will allow clinicians to individualize therapies based on specific tumor characteristics. In one recent open‐label randomized trial, investigators found that individuals with the ABC subtype of DLBCL who were treated with rituximab, doxorubicin, cyclophosphamide, vindesine, bleomycin, and prednisone (R‐ACVBP) exhibited improved survival as compared to those who received R‐CHOP. Notably, there was no significant difference in survival among those with the GCB subtype.^25^ Other recent studies have demonstrated that the ABC subtype of DLBCL responds poorly to immunotherapy, whereas immunotherapy is generally effective for the management of the GBC subtype.^26^ Lenalidomide also appears to be effective for the management of refractory cases of the ABC subtype of DLBCL, but not the GCB subtype.^27^ Indeed, it appears that further genetic characterization of DLBCL will lead to more effective, individualized therapies in the years to come.

## 4 CONCLUSION

Diffuse large B‐cell lymphoma is one of the most common hematologic malignancies in the United States. The classic presenting feature is local or generalized lymphadenopathy. However, extranodal manifestations are common and may involve the central nervous system, gastrointestinal tract, or, as in our patient, the musculoskeletal and genitourinary systems. Shoulder pain and stiffness are particularly worrisome for malignancy. A careful physical examination with appropriate follow‐up laboratory and imaging studies can help establish an early diagnosis. Treatment typically involves chemotherapy, which can be individualized based on DLBCL subtype.

## CONFLICT OF INTEREST

None declared.

## AUTHOR CONTRIBUTION

BDB: wrote the case report and edited and finalized the manuscript. RK: wrote and edited the portions of the manuscript on imaging and pathology. PP: wrote and edited the literature review and treatment section of the manuscript. MBU: conceived of the project and helped with language editing. CLE: involved in the case and provided technical editing. JF: involved in the case and provided technical editing. RI: proofread the final draft of the manuscript. NG: supervised the project from initiation to completion.

## References

[1] Morton LM , Wang SS , Devesa SS , Hartge P , Weisenburger DD , Linet MS . Lymphoma incidence patterns by WHO subtype in the United States, 1992–2001. Blood. 2006;107(1):265‐276. 16150940 10.1182/blood-2005-06-2508 PMC1895348

[2] López‐Guillermo A , Colomo L , Jiménez M , et al. Diffuse large B‐cell lymphoma: clinical and biological characterization and outcome according to the nodal or extranodal primary origin. J Clin Oncol. 2005;23(12):2797‐2804. 15728226 10.1200/JCO.2005.07.155

[3] SEER . Diffuse Large B‐Cell Lymphoma – Cancer Stat Facts. 2016 http://seer.cancer.gov/statfacts/html/dlbcl.html. Accessed August 16, 2019.

[4] Shankland KR , Armitage JO , Hancock BW . Non‐Hodgkin lymphoma. Lancet. 2012;380(9844):848‐857. 22835603 10.1016/S0140-6736(12)60605-9

[5] Pedersen AB , Horváth‐Puhó E , Ehrenstein V , Rørth M , Sørensen HT . Frozen shoulder and risk of cancer: a population‐based cohort study. Br J Cancer. 2017;117(1):144‐147. 28524156 10.1038/bjc.2017.146 PMC5520209

[6] Jawad MU , Schneiderbauer MM , Min ES , Cheung MC , Koniaris LG , Scully SP . Primary lymphoma of bone in adult patients. Cancer. 2010;116(4):871‐879. 20043324 10.1002/cncr.24828

[7] Lim CY , Ong KO . Imaging of musculoskeletal lymphoma. Cancer Imaging. 2013;13(4):448‐457. 24334414 10.1102/1470-7330.2013.0036 PMC3864222

[8] Krishnan A , Shirkhoda A , Tehranzadeh J , Armin AR , Irwin R , Les K . Primary bone lymphoma: radiographic–MR imaging correlation. Radiographics. 2003;23(6):1371‐1387. 14615550 10.1148/rg.236025056

[9] Graham TS . The ivory vertebra sign. Radiology. 2005;235(2):614‐615. 15858100 10.1148/radiol.2352021743

[10] Hwang S . Imaging of lymphoma of the musculoskeletal system. Radiol Clin North Am. 2008;46(2):379‐396. 18619386 10.1016/j.rcl.2008.03.008

[11] Li S , Young KH , Medeiros LJ . Diffuse large B‐cell lymphoma. Pathology. 2018;50(1):74‐87. 29167021 10.1016/j.pathol.2017.09.006

[12] Crombie JL , Armand P . Diffuse large B‐cell lymphoma and high‐grade B‐cell lymphoma: genetic classification and its implications for prognosis and treatment. Hematol Oncol Clin North Am. 2019;33(4):575‐585. 31229155 10.1016/j.hoc.2019.03.001

[13] Abramson JS . Hitting back at lymphoma: how do modern diagnostics identify high‐risk diffuse large B‐cell lymphoma subsets and alter treatment? Cancer. 2019;125(18):3111‐3120. 31287161 10.1002/cncr.32145

[14] Chapuy B , Stewart C , Dunford AJ , et al. Molecular subtypes of diffuse large B cell lymphoma are associated with distinct pathogenic mechanisms and outcomes. Nat Med. 2018;24(5):679‐690. 29713087 10.1038/s41591-018-0016-8 PMC6613387

[15] Liu SZ , Zhou X , Song A , et al. Successful management of a rare case of humerus non‐Hodgkin's lymphoma in rapid progress. Chin Med J (Engl). 2018;131(14):1753‐1754. 29998900 10.4103/0366-6999.235886 PMC6048933

[16] Caporale MF , Gambino GF , Larosa FS , Del Buono A , Di Segni F . Non‐Hodgkin's lymphoma: unexpected cause of shoulder pain. A systematic review of the literature. Muscles Ligaments Tendons J. 2013;3(3):236‐239. 24367786 PMC3838335

[17] Hatem J , Bogusz AM . Bogusz AM: An unusual case of extranodal diffuse large B‐cell lymphoma infiltrating skeletal muscle: a case report and review of the literature. Case Rep Pathol. 2016;2016:1‐8. 10.1155/2016/9104839 PMC4877472 27247818

[18] Tuck M , Lim J , Lucar J , Benator D . Anaplastic large cell lymphoma masquerading as osteomyelitis of the shoulder: an uncommon presentation. BMJ Case Rep. 2016 10.1136/bcr-2016-217317 PMC5237804 28003232

[19] Kishan Prasad HL , Jayprakash Shetty K , Mathias L , et al. Primary bone lymphoma of the humerus diagnosed by FNAC – a rare case report. Indian J Surg Oncol. 2013;4(3):316‐319. 24426747 10.1007/s13193-013-0251-x PMC3771065

[20] Liu Y‐C , Chang W‐L , Gau J‐P , Chao T‐C . Primary bone lymphoma of the shoulder. Br J Haematol. 2012;158(6):677. 22812585 10.1111/j.1365-2141.2012.09235.x

[21] Pitman SD , Liwnicz B , Wang J . Pathologic quiz case: a 70‐year‐old woman with long‐standing shoulder pain. Arch Pathol Lab Med. 2003;127(7):885‐886. 12823050 10.5858/2003-127-885-PQCAYO

[22] Moazzam N , Malik AA , Potti A . B‐cell lymphoma mimicking multiple myeloma. Leuk Lymphoma. 2002;43(9):1869‐1873. 12685847 10.1080/1042819021000006556

[23] Bohgaki T , Notoya A , Mukai M , et al. CD‐30 positive anaplastic large cell lymphoma of the bone treated with autologous peripheral blood stem cell transplantation. Rinsho Ketsueki. 1999;40(12):1252‐1257. 10658478

[24] Schneider C , Pasgualucci L , Dalla‐Favera R . Molecular pathogenesis of diffuse large B‐cell lymphoma. Semin Diagn Pathol. 2011;28(2):166‐177. 10.1053/j.semdp.2011.04.001 PMC3562715 21842702

[25] Molina TJ , Canioni D , Copie‐Bergman C , et al. Young patients with non‐germinal center B‐cell‐like diffuse large B‐cell lymphoma benefit from intensified chemotherapy with ACVBP plus rituximab compared with CHOP plus rituximab: analysis of data from the Group d’Etudes des Lymphomes de l’Adulte/lymphoma study associated phase III trial LNH 03–2B. J Clin Oncol. 2014;32(35):3996‐4003. 25385729 10.1200/JCO.2013.54.9493

[26] Nowakowski GS , Czuczman MS . ABC, GCB, and double‐hit diffuse large B‐cell lymphoma: does subtype make a difference in therapy selection?. Am Soc Clin Oncol Educ Book. 2015;35:e449‐457. 10.14694/EdBook_AM.2015.35.e449 25993209

[27] Hernandez‐Ilizaliturri FJ , Deeb G , Zinzani PL , et al. Higher response to lenalidomide in relapsed/refractory diffuse large B‐cell lymphoma in nongerminal center B‐cell‐like than in germinal center B‐cell‐like phenotype. Cancer. 2011;117(22):5058‐5066. 21495023 10.1002/cncr.26135

